# Heavy Metals in Agricultural Soils: Sources, Influencing Factors, and Remediation Strategies

**DOI:** 10.3390/toxics12010063

**Published:** 2024-01-12

**Authors:** Yanan Wan, Jiang Liu, Zhong Zhuang, Qi Wang, Huafen Li

**Affiliations:** Beijing Key Laboratory of Farmland Soil Pollution Prevention and Remediation, Key Laboratory of Plant-Soil Interactions of the Ministry of Education, College of Resources and Environmental Sciences, China Agricultural University, Beijing 100193, China; wanyanan@cau.edu.cn (Y.W.); 15548810038@163.com (J.L.); zhuangzhong@cau.edu.cn (Z.Z.); wangqi88@cau.edu.cn (Q.W.)

**Keywords:** agricultural soil, heavy metal, source, safe utilization, remediation

## Abstract

Soil heavy metal pollution is a global environmental challenge, posing significant threats to eco-environment, agricultural development, and human health. In recent years, advanced and effective remediation strategies for heavy metal-contaminated soils have developed rapidly, and a systematical summarization of this progress is important. In this review paper, first, the anthropogenic sources of heavy metals in agricultural soils, including atmospheric deposition, animal manure, mineral fertilizers, and pesticides, are summarized. Second, the accumulation of heavy metals in crops as influenced by the plant characteristics and soil factors is analyzed. Then, the reducing strategies, including low-metal cultivar selection/breeding, physiological blocking, water management, and soil amendment are evaluated. Finally, the phytoremediation in terms of remediation efficiency and applicability is discussed. Therefore, this review provides helpful guidance for better selection and development of the control/remediation technologies for heavy metal-contaminated agricultural soils.

## 1. Introduction

Soil is the foundation of agricultural production, which contains a wide range of mineral elements, including essential trace elements and toxic elements. Among them, Zn and Cu, etc. are essential for healthy functions in humans, animals, and plants in appropriate dosages, but they can be harmful at excess concentration; whereas, Cd, Hg, and As, etc. are non-essential and toxic to organisms, even at low concentrations [1,2]. However, these toxic elements can be absorbed by plant roots due to the similarities of their physiochemical properties to essential elements [3]. Over the last decades, with the rapid development of industrialization and overpopulation, a large number of heavy metals have been produced and entered the soil through anthropogenic interference, and soil heavy metal pollution has become a global issue. It was reported that over five million sites worldwide have been contaminated with heavy metals [4], and in some countries such as China, India, and Egypt, a considerable portion of soil failed to meet the soil quality standard limits for Cd, As, and Pb, etc. [5,6,7].

Heavy metal contamination of soil could impair ecosystems and human health by direct contact with contaminated soil and via the ingestion of food from contaminated soil. Under toxic metal stress, the physiological processes of plants could be disrupted, such as the damage of cell membrane integrity, the increase in oxidative damage, the disbalance of essential nutrient uptake, the reduction in photosynthetic activity, and the inhibition of plant morphology and physiology [2]. Furthermore, toxic heavy metals taken up by plant roots present risks not only to crops but also to the consumers. According to the concept of “soil-plant barrier” by Chaney [8], some elements, such as Cd and As, have relatively high solubility and mobility in soil, high transport capacity in plants, and are toxic to humans and animals at low levels, such as renal failure and lung and skin cancers [9]. In some regions, some crops such as rice and vegetables have continually failed to meet the food quality standard limits, posing a great risk to the residents living in heavily metal-contaminated areas who mainly consume locally produced crops [7,10,11].

In the last several decades, different strategies based on physical, chemical, and biological methods have been implemented to reduce heavy metal accumulation in crops, and the systematic summarizing of these strategies is essential. The present paper introduces the sources of heavy metals in agricultural soils, the factors affecting heavy metal accumulation in crops, and the strategies to reduce heavy metal accumulation in crops. This review will provide helpful guidance for better selection and development of control/remediation technologies for heavy metal-contaminated agricultural soils.

## 2. Sources of Heavy Metal in Agricultural Soils

**Figure 1:**
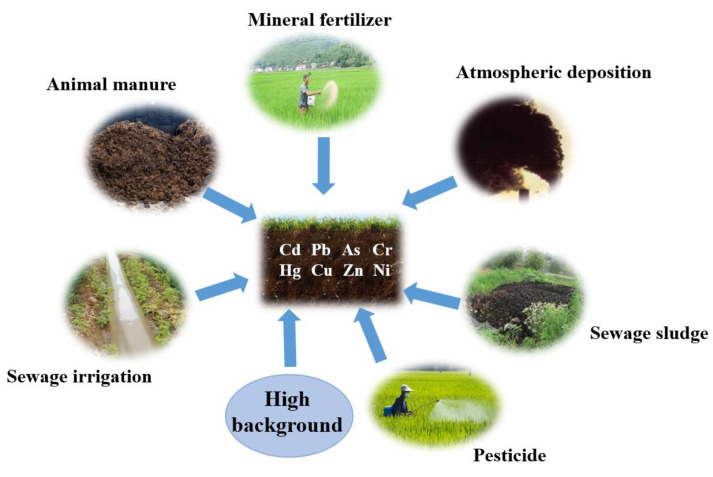
Main sources of heavy metals in agricultural soils.

Heavy metals enter the soil in two ways: natural activities and anthropogenic activities. Natural activities include pedogenic processes (high background) and volcanoes and forest fires; anthropogenic sources include mining, smelting, transportation, and agricultural activities, which are considered the major causes of heavy metal contamination in soil. Besides high background regions, the main inputs of heavy metals to agricultural lands are atmospheric deposition, sewage irrigation, sewage sludge, animal manure, mineral fertilizers, and pesticides (Figure 1), among which, atmospheric deposition is considered the predominant contributor to agricultural soils, especially in more industrial countries such as China [12] and the UK [13]. According to the reports by Luo et al. [12] and Ni et al. [14], atmospheric deposition accounted for 43–85% of the total inputs of As, Cr, Hg, Ni, and Pb in agricultural soils in 1999–2005, and the proportions increased to 80–94% in 2006–2015 in China. On the other hand, animal manures were responsible for 55%, 69%, and 51% of the total Cd, Cu, and Zn inputs, respectively, during 1999–2005; and for 20%, 63%, and 44%, respectively, during 2006–2015 [12,14]. However, in France, animal manures, mineral fertilizers, and pesticides are the predominant sources of heavy metals in farmland. For example, over 50% of the total Zn, Ni, As, Cu, and Hg that reached agricultural lands were derived from animal manures, and only less than 20% were derived from atmospheric deposition; whereas about 50% of Cd and Cr inputs were from mineral fertilizers [15]. 

Previous studies have shown that heavy metal contaminants, such as Pb and Hg, in agricultural soils in some industrial areas are largely related to coal combustion, industrial discharge, and vehicle emissions via atmospheric deposition. Peng et al. [16] reported that the contribution rates of coal combustion and vehicle emissions to Pb in atmospheric dust were 22% and 78%, respectively, in Northeast China. Animal manures are regarded as valuable fertilizers, but they are also the main sources of heavy metal input into the soil. To improve weight gains (or egg production) and prevent disease, heavy metal feed additives (such as As9, Cu, and Zn) are used in intensive animal production systems, leading to an increase in these heavy metals in manure by-products [17]. Our previous study found that the average contents (median) of Cd, As, Cu, and Zn in animal manures were 2.3 (0.72), 14.0 (3.5), 282 (115), and 656 (366) mg kg^−1^, respectively, in China [18]. The continuous application of chicken or pig manure caused the accumulation of heavy metals in paddy soil, with accumulation rates of 0.006–0.026 and 0.002–0.025 mg kg^−1^ yr^−1^ for Cd, 0.15–1.2 and 1.0–4.2 mg kg^−1^ yr^−1^ for Cu, and 0.54–5.5 and 1.5–9.6 mg kg^−1^ yr^−1^ for Zn, respectively [19,20]. Phosphate fertilizer is also considered an important source of heavy metals, especially Cd, which originates from phosphate rocks. According to 196 phosphate fertilizer samples from 12 European countries, the average heavy metal contents in the fertilizers were 7.4 (Cd), 90 (Cr), 166 (Zn), 15 (Ni), and 2.9 (Pb) mg·kg^−1^, respectively [21], with higher input fluxes of Cr and Cd from phosphate fertilizers than from atmospheric deposition [15,21]. And the Cd contents in phosphate fertilizers in America [22] and Brazil [23] were 5–41 and 0.14–51 mg kg^−1^, respectively. In China, however, the phosphorus fertilizer contained much less Cd, varying from N.D. to 27 mg kg^−1^, with an average content of 0.91 mg kg^−1^ [24]. Pesticide is usually a significant input of Cu in some agricultural lands; for example, in France, over 30% of the total Cu in agricultural land is from pesticides [15] because of the increased application of Cu-containing pesticides (such as the Bordeaux mixture) on vineyard and orchards. Sewage sludge and sewage irrigation were much smaller than those from atmospheric deposition and fertilization [13,14], but could also be the sources of heavy metal contamination, where these materials were excessively applied. For example, in Hunan province in southern China, due to the contamination of Xiangjiang, the Cd input from irrigation could reach 1–400 g hm^−2^ yr^−1^ [5]. Therefore, policies should be developed to control toxic heavy metal input and accumulation in agricultural soils.

## 3. Factors Affecting Heavy Metal Accumulation in Crops

**Figure 2:**
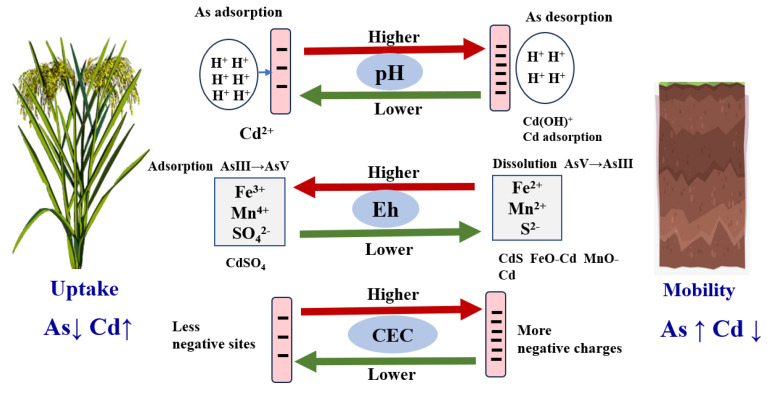
Mechanisms of heavy metal mobility in the soil are affected by soil factors.

The uptake and accumulation of heavy metals in plants depend both on plant physiological features and soil components, including soil pH, redox potential (Eh), organic matter content (OM), clay content, and cation exchange capacity (CEC) (Figure 2).

### 3.1. Plant Species/Cultivars

Great differences in metal absorption, translocation, and distribution among and within plant species have been observed, even when planted in the same contaminated site. Generally, leafy, stem, and bulb vegetables accumulate higher contents of heavy metals (such as Cd, Cu, and Pb) in their edible parts than melon, fruit, and bean vegetables [25,26]. A field study showed that the bioconcentration factor (BCF) of Cd in leafy vegetables, stem and bulb vegetables, solanaceous vegetables, and bean vegetables were 0.12, 0.17, 0.04, and 0.03, respectively [26]. Large genetic variations in heavy metal accumulation still exist among the cultivars; for example, the ability to accumulate Cd is higher in *indica* than in *japonica* varieties [27]. And this is one of the possible reasons for the relatively high rice Cd content in South China, since the rice varieties grown in South China are mainly *indica* types, whereas the varieties planted in Middle and Northern China are usually *japonica* types. It has been reported that the median values of Cd content in polished rice produced in North, East, Middle, and South China regions were 0.005, 0.028, 0.052, and 0.11 mg·kg^−1^, respectively [28]. The difference in Cd accumulation in rice grains between *indica* and *japonica* could be attributed to the natural missense mutation Val449Asp in OsCd1 (involved in Cd uptake and grain accumulation in rice) in *indica* [29]. 

Heavy metal tolerance and detoxification are responsible for the divergence in heavy metal accumulation in plants. Root exudates, such as organic acids from rhizospheric microbes and phytosiderophores, can activate metals (such as Cd) in the rhizosphere, facilitating their uptake by plants [30]. Once in the plant, the root cell wall can provide some functional groups to join metal ions together and restrain their entry into the cytomembrane, which is the first barrier to toxic ions. In plant cells, heavy metals usually bind to sulfur ligands (e.g., glutathione (GSH), phyochelatins (PCs)), and the heavy metals-PCs complexes are then transferred into vacuoles via vesicular membrane ATPase, resulting in the less translocation of metals from root to shoot [31]. According to the results from 28 Chinese kale cultivars, the proportions of Cd distributed to the cell wall and vacuole of root were higher in a typical low-Cd cultivar than in a typical high-Cd cultivar [32], leading to less Cd root-to-shoot translocation, which is considered an important mechanism of cultivar-dependent Cd accumulation in plants. The root-to-shoot translocation is also affected by the transpiration capacity of plants; our previous study showed that the Cd contents in the edible parts of 18 pakchoi cultivars grown in Cd-contaminated soil (3.6 mg·kg^−1^) ranged from 0.33 to 1.6 mg kg^−1^ in the summer, and the shoot Cd content was positively correlated with the transpiration rate of pakchoi [33]. 

### 3.2. Soil Components

Heavy metals in the soil exist in various forms, and not all of them are available for plants. Soil pH is considered the key factor controlling the solubility and mobility of heavy metals in soil, and thereby their uptake by plants. In several studies, the availability of cationic metals in plants was negatively correlated with soil pH [34]. As the soil pH increases, the negative charge on the surface of soil colloids increases, resulting in the formation of more Fe and Mn oxides in soil and consequently an increase in adsorption capacity towards the metal. In addition, M(OH)^+^, can be formed through hydrolysis, which can be easily adsorbed by soil colloids [35]. In contrast, with an increase in soil pH, the consumption of protons can enhance the desorption of As, leading to an increased release of As into the soil solution [36]. In paddy soil, soil redox potential (Eh) related to water management essentially controls metal solubility. Under flooding conditions (at a reduction potential), Fe(III), Mn(IV/VI), and SO_4_^2−^ are reduced to Fe^2+^, Mn^2,+,^ and S^2−^, by receiving electrons produced by the respiration of microbes (such as sulphate-reducing bacteria) in soils, Cd^2+^ in soil forms Fe/Mn oxide-bound Cd [37], and CdS [38]. However, the availability of As in the soil increases under reducing conditions as a result of the dissolution of iron (oxyhr)oxides and the reduction of arsenate (AsV) to arsenite (AsIII), which is more mobile and toxic [39,40,41].

Soil organic matter is another important soil characteristic that influences the availability of heavy metals in soil, which can immobilize heavy metals through adsorption or the formation of a stable organic fraction. It has been established that a large number of functional groups (e.g., COOH^−^ and OH^−^) exist in humic substances [42], which play an important role in forming complexes with metal ions, thereby reducing the metal movement in soil [43]. Numerous studies showed a reduction effect of humic substances on the heavy metal uptake by plants [44,45]. However, organic matter can also supply organic chemicals to the soil solution, which can serve as chelates and enhance the Cd availability to plants [46]. These contradictory results may be due to the different soil types, experimental conditions, sources, and application rates of organic matter, which makes it hard to obtain consistent conclusions. Other soil characteristics such as CEC and clay content also play vital roles in deducing the availability of metals. When CEC is high, a high amount of Cd is stabilized in soil colloids, whereas Cd in the soil solution is high at low CEC [47]. In addition, clay particles with negatively charged surfaces can adsorb heavy metals, and the quantities of Pb, Cd, Zn, and As are higher in fine soil particles than in coarser particles [48].

## 4. Strategies to Reduce Heavy Metal Accumulation in Crops

**Figure 3:**
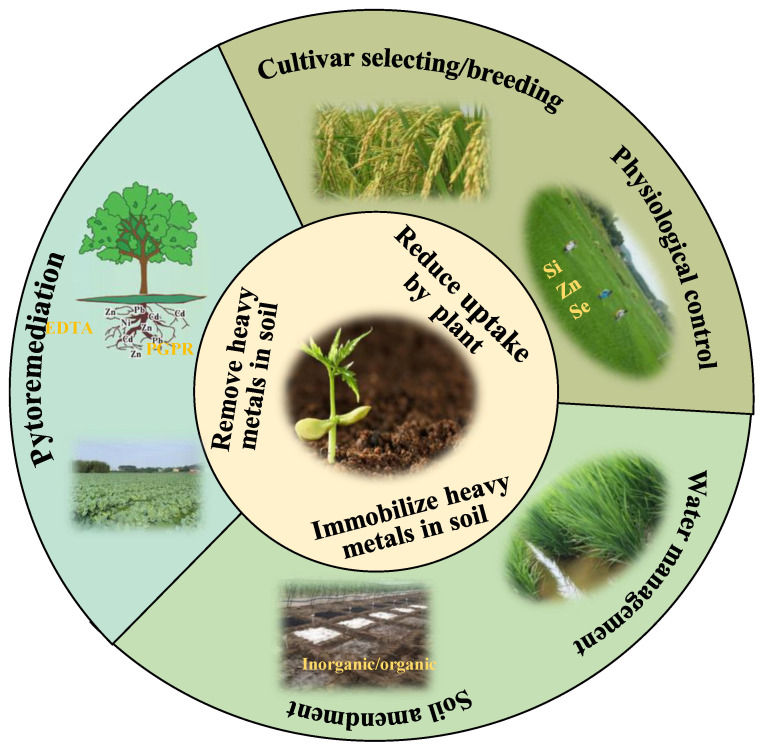
Measures for reducing heavy metal accumulation in crops.

To minimize heavy metal exposure to humans, effective measures should be taken to reduce the uptake of heavy metals by crops and immobilize or remove heavy metals from the soil. These measures mainly include (1) low-metal cultivar selection/breeding; (2) physiological blocking; (3) water management; (4) soil amendment; (5) phytoremediation (Figure 3). 

### 4.1. Low-Metal Cultivars Selecting/Breeding

To reduce the entry of heavy metals into the food chain, alternative food crops or cash crops with low heavy metal accumulation could be planted. However, alternative planting has not been commonly practiced, especially on large scales, due to the difficulties farmers face in changing their agricultural habits. Nevertheless, low-metal cultivars are proposed as an economical and environmentally friendly approach to address the low-to-medium heavy metal-contaminated farmland. To date, the pollution-safe cultivar strategy has been utilized in various crops; for example, among 72 popular wheat genotypes in the Huang-Huai-Hai Plain in China, nine wheat cultivars with stable low-Cd and moderately high microelements contents in grain were identified and tested based on three different farmlands with various Cd contents and soil properties, and the nine cultivars were recommended for planting in slightly-to-moderately Cd-contaminated soil [49]. In a study by Li et al. [27], the BCF of grain Cd in 183 rice cultivars was divided into five grades: ultra-low accumulation, low accumulation, moderate accumulation, high accumulation, and ultra-high accumulation, by combining an empirical soil–plant transfer model with species sensitivity distribution (SSD), which provided a method for identifying safe cultivars in contaminated soil. Selecting low-metal accumulation cultivars from existing commercial cultivars with excellent agronomic traits and high yield can be directly used in production, but it usually requires a lot of effort and a time-consuming selection process. 

In recent decades, the genes involved in heavy metal uptake, translocation, and detoxification have been identified in plants, so molecular breeding has been widely used in approaches to reduce heavy metal accumulation in plants directly. In rice, Cd is taken up by rice roots primarily via the metal transporters’ natural resistance-associated macrophage proteins (NRAMPs), for example, OsNramp5, a manganese transporter located at the cytoplasma membrane of rice, while heavy metal ATPases (HMAs) play direct roles in transmembrane transport of heavy metals; for example, OsHMA3 plays an important role in mediating the transport of Cd from the cytoplasm into the vacuole, and OsHMA2 is involved in the delivery of Cd to developing tissues [50,51]. The knockout or knockdown of OsNramp5 can reduce Cd uptake and accumulation in rice; however, it might also cause yield losses due to Mn deficiency [52], and genetic modification is not commonly accepted by the public. In a study by Ishikawa et al. [50], three rice mutants (lcd-kmt1, lcd-kmt2, and lcd-kmt3) with <0.05 mg kg^−1^ Cd in the grain were produced by carbon ion-beam irradiation, compared with a mean of 1.7 mg kg^−1^ Cd in the parent (Koshihikari), and the yield of lcd-kmt1 was not reduced, which was widely accepted by the public. The overexpression of OsHMA3 resulted in a more than 90% decrease in the Cd content of brown rice, but it did not significantly affect grain yield and the content of other micronutrients (e.g., Zn, Fe, Cu, and Mn) [51]. In addition, low-affinity cation transporter 1 (OsLCT1), which is expressed in the nodes, has been identified as a transporter for transporting Cd from leaf blades and nodes into grains. Thus, the downregulation of OsLCT1 decreased Cd in rice grain by about 50%, while little effect was found on the plant growth and the content of other metals [53]. As for arsenic, the characterization of the Si influx/efflux transporter (Lsi1 and Lsi2) [54] and the ATP-binding cassette transporter [55] creates many possibilities for developing low-As cultivars. With the development of genomics technologies, molecular breeding for low-metal accumulation cultivars with high quality and yield has received increasing interest, and the integration of molecular breeding with conventional breeding methods will accelerate the development of breeding low-heavy metal cultivars. 

### 4.2. Physiological Blocking 

Mineral nutrients are essential in the process of gene expression, photosynthesis, metabolism, and other activities of plants, and the deficiency of macro- or micro-elements could lead to a negative influence on plant growth and development. Meanwhile, mineral elements can also alleviate heavy metal accumulation and toxicity in plants by alleviating oxidative stress, restoring cell membrane integrity, enhancing photosynthesis, balancing the uptake of essential nutrients, and regulating the uptake, translocation, distribution, and speciation of heavy metals in plants [31,56]. In recent years, many mineral fertilizers (e.g., Si, Se, and Zn) have been reported to effectively decrease heavy metal (e.g., Cd, As, and Hg) accumulation in the crop, such as rice [57], wheat [58], and maize [59]. 

**Figure 4:**
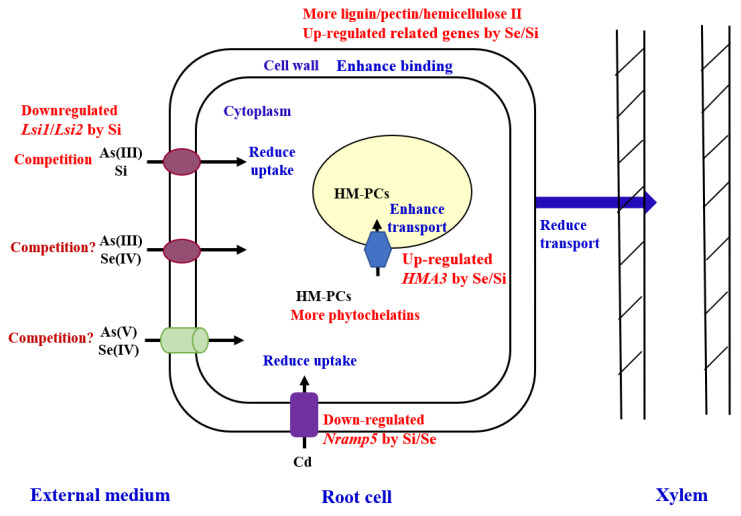
Mechanisms of Si/Se in regulating heavy metal uptake, transport, and detoxification in plants.

Cell wall binding and vacuolar sequestration are effective mechanisms of heavy metal detoxification. And the antagonism of Si/Se on the accumulation and toxicity of heavy metals is mainly related to these processes (Figure 4). Studies have claimed that Si/Se could upregulate the expression of genes involved in the synthesis of lignin and increase the formation of lignin [60,61,62], as well as enhance the binding capacity of the cell wall by increasing the formation of pectin and hemicellulose II [63]. Moreover, glutathione peroxidase (GSH-Px) can be activated by Se under heavy metal stress, and Se can promote the production of PCs, which are involved in metal detoxification and sequestration [64]. Se/Si addition increased the Cd sequestrated in the vacuole [65] and upregulated the expression of genes (OsHMA3) responsible for the process [59,60]. As a result, the sequestration and detoxification of Cd in cells results in less root-to-shoot Cd translocation in plants. In a study by Gao et al. [57], foliar spraying with Si (2.5 mmol/L) or Se (40 mg/L) at the tillering and heading stages significantly decreased Cd in brown rice in a high Cd translocation cultivar by 72% and 62%, respectively. Xia et al. [66] reported that the foliar application of Se significantly reduced Cd in wheat grain by 32% by reducing the translocation of Cd from flag leave to the grain, and the application at the filling stage was better in reducing Cd content in grains than application at the heading stage. Moreover, it was also shown that Si/Se could decrease the expression of OsNramp5 in rice [60,61] and reduce the uptake of Cd by rice. In addition, Si could decrease the As uptake and transport in plants through the competition for binding sites on their transporters (e.g., Lsi1 and Lsi2) and the downregulation of related genes (e.g., Lsi1 and Lsi2), since As transport in rice roots shares the same highly efficient pathway as Si [54]. Se ameliorated As toxicity via the upregulation of GST (glutathione S-transferase), PRX (peroxidase), and GRX (glutaredoxin) [67]. In previous studies, the root application of Se or Si could simultaneously reduce Cd and As accumulation in rice grain [68,69]. In addition, Se could combine with inorganic Hg to form insoluble HgSe (or/and HgSe containing proteinaceous) complexes both in the soil and plants and reduce the bioavailability of Hg in the soil and the translocation in plants [70,71].

Due to their similar physical–chemical and environmental characteristics, Zn and Cd interact with each other in soil–plant systems. During the process of uptake and translocation in plants, Zn could compete with Cd for binding sites on the transporters related to metal uptake and translocation, and it could also regulate the expression of related genes [72]. In addition, Zn can reduce Cd accumulation in plants by regulating the antioxidant system and sequestering Cd into the cell wall of leaves [56]. Field studies showed that foliar application of Zn (0.3–0.5% ZnSO_4_·7H_2_O) at the filling stage significantly reduced Cd in rice grain by 21–41% [31,57]. And foliar application of 0.3% ZnSO_4_·7H_2_O effectively reduced Cd in wheat grain [73], and foliar spraying at the booting stage was the most suitable time to minimize the Cd-induced loss in grain yield and decrease grain Cd content [58].

The effect of the combined application of different fertilizers on reducing heavy metal accumulation in cereals is still not clear. In a study by Huang et al. [74], a strong synergistic effect of Si and Se was found in alleviating Cd toxicity to rice by regulating gene expression, sequestering Cd into the root cell walls and vacuoles, and decreasing Cd translocation from the root to the shoot. Similarly, the synergistic effect of Si and Zn was observed in alleviating Cd toxicity by improving the ascorbate–glutathione cycle and antioxidant enzyme activities [75]. However, field studies showed that the combined foliar application of Si and Se was less effective for decreasing Cd in rice grain than the application of Si or Se individually, possibly due to the large colloidal particle size in the combined treatment [57,76].

### 4.3. Water Management

Water management affects heavy metal bioavailability by influencing the soil Eh and pH. As mentioned in Section 3.2, flooding (at a reduction potential) decreased the availability of cationic metals (such as Cd and Pb) in soil; and in acidic soils, the reduction reactions could consume H^+^, causing a rise in pH. And the rise in pH enhances the metal adsorption to the soil by increasing the formation of M(OH)^+^ [34]. Additionally, flooding can affect metal accumulation in rice by affecting the formation of iron plaque, the radial oxygen loss (ROL) barrier, and the mass flow in rice [77]. Our previous study showed that flooding significantly reduced Cd and Pb accumulation while aerobically increasing Cd and Pb levels in rice grain [78]. And in a study by Mlangeni [79] and Liu [80], the Cd in brown rice under continuous flooding was higher than that of wetting irrigation or alternate wetting and drying. For Pb, alternate wetting and drying have been demonstrated to effectively reduce Pb accumulation in rice compared to continuous ponding [81]. The effect of flooding on the bioaccumulation of metals in rice is also related to soil properties, and in our previous study, flooding reduced the Cd in rice grain by 92% in acidic soil, while minor changes were found in slightly alkaline soil [82]. Overall, water management would be a practical and inexpensive method to reduce Cd accumulation in rice in a single Cd-contaminated land.

However, the contrasting biogeochemical behaviors of Cd and As result in different effects of water management on their accumulation in rice grain. Under flooding conditions, the rice has low Cd but high As in the grain; whereas in aerobic soil, the rice has high Cd but low As in the grain [78,83]. Therefore, the optimization of the trade-off relationship between Cd and As is needed. In a study by Bao et al. [84], a combined treatment of 5 days of flooding + 3 days of drainage (repeated every 8 days from the elongation stage to the flowering stage) was identified as the most effective treatment for decreasing grain Cd and As simultaneously. The adoption of sprinkler irrigation greatly minimizes the Cd (−50% relative to continuous flooding), Pb (−50%), and As (−98%) contents in 26 rice cultivars [85]. And according to Honma et al. [34], for minimizing the trade-off relationship between Cd and As in rice grain, the optimal soil pH of 6.2 and Eh of −73 mV should be targeted during the 3 weeks after heading, by appropriate water management. In fact, water management applied at different growth stages had significantly different influences on rice grain Cd and As contents. Huang et al. [86] found that 98% of the Cd in rice grain was derived from root uptake during the grain filling period, while 95% of the grain As was derived from the remobilization of As in other parts previously accumulated within vegetative growth periods; as a result, water management at filling stage had a much more obvious effect on Cd than As in rice grain, while water management during the vegetative growth period had a greater effect on As. Therefore, a segmented water management strategy can be designed to control Cd and As in rice grain simultaneously. 

### 4.4. Soil Amendment

In situ immobilization and stabilization are considered economical and effective techniques to reduce the bioavailability of heavy metals in contaminated soil. In recent decades, inorganic and organic soil amendments, such as lime, phosphate minerals, clay minerals, biochar, and livestock manure, have been widely used for soil control and remediation (Table 1). The essential mechanisms of heavy metal immobilization by soil amendments are related to adsorption, complexation, cation exchange, and precipitation.

Commonly, the solubility and mobility of cationic metals are higher at low pH, whereas lime can increase soil pH by releasing hydroxyl ions, thus decreasing the bioavailability of metals (such as Cd and Pb). And the application of lime enhances competition between calcium and Cd, reducing the uptake by plants [87]. Phosphorous-containing materials could immobilize heavy metals such as Cd and Pb in soil by increasing soil pH, electrostatic interaction, ion exchange, and surface complexation while improving soil fertility through the release of phosphorus [88]. Clay minerals, including sepiolite, attapulgite, bentonite, and kaolinite, have large specific surface areas for excellent adsorbents, and the metal cation might be immobilized through outer-sphere complex, inner-sphere complex, lattice diffusion, and isomorphic substitution within the mineral lattice [89]. In a study by Garau et al. [87], the addition of lime, zeolite, or red mud significantly reduced the solubility of Cd, Pb, and Zn in a combined-contaminated acidic soil, while the increase in soil pH was identified as a common mechanism of action for lime and red mud. When treated with lime or red mud, the soil pH increased from 4.23 to 7.11 and 7.14, respectively. And Bashir et al. [90] found that the addition of 3% or 5% sepiolite increased the soil pH by 0.8 and 1.0 units, decreased the acid-soluble Cd content by 33% and 43%, and decreased Cd content in spinach shoots by 11.2% and 26.2%, respectively. In fact, sepiolite, bentonite, and palygorskite are alkaline materials, which would have better effects for the treatment of Cd-contaminated acidic soils.

Manures and composts (based on animal manures and/or plants) are considered the cost-effective and environmentally friendly ways to dispose and recycle waste, which can not only maintain the soil capability and provide nutrients to plants but also reduce the mobility and bioavailability of heavy metals in soil through complexation, adsorption, and precipitation [91]. In our previous study, the application of chicken or swine manure significantly reduced extractable Cd and Pb in the soil, and significantly reduced the Cd and Pb contents in rice grain by 7.8–79% and 7.2–59%, respectively, with increasing application rates and number of years [19]. However, livestock manure is also considered an important source of heavy metal contamination in the soil. A 15-year protected field vegetable production experiment showed that the continuous application of manure increased the total and available Cd, Cu, and Zn in the soil, thereby increasing their potential risk in vegetables [92]. Thus, effective strategies should be adopted to strengthen the quality regulation of organic fertilizers. 

Biochar, which is obtained from the combustion process (around 200 to 700 °C) by using crop residues, woods, etc., is a stable compound with high contents of carbon and macro-elements, and high adsorption capacity. Biochar can affect metal mobility and bioavailability in soils directly through cation-π interactions, electrostatic attraction, ion exchange, complexation, and precipitation, and indirectly by influencing soil characteristics such as pH, organic carbon, CEC, and minerals [93]. In some pot or field trials, biochar application obviously reduced the mobility of heavy metals in soils and their accumulation in plants, and promoted crop yields; however, different results were also found following biochar amendment, which could be due to the various soil types, raw materials of biochar, and application rate. [88,93]. Furthermore, the adsorption capacity of biochar differs with pyrolysis temperature; for example, compared to 200 °C, soybean straw, sewage sludge, or peanut shells derived from biochar produced at 350 °C significantly reduced the availability of Cd and As in soil and their subsequent accumulation in rice plants [94]. 

To enhance the remediation performance of soil amendments and resolve the difficulty of combined contamination, various combined/modified (e.g., functional groups, encapsulation) methods were adopted in their production or application, such as Fe/Zn-modified biochar, goethite-modified biochar, and mercapto-modified palygorskite. For example, among the treatments of single biochar, single goethite, goethite-combined biochar, and goethite-modified biochar, the latter two could effectively immobilize Cd and As simultaneously, and the goethite-modified biochar treatment was the optimum additive, with the immobilization efficiency of 57% (CaCl_2_-Cd) and 61% (TCLP-As, Toxicity Characteristic Leaching Procedure) for Cd and As, respectively [95]. Both Cd and As in rice plants were significantly decreased by the zero-valent iron-biochar treatment, and the decreases in rice grain were 93% and 61%, respectively [96]. In addition, some functional materials, such as nanomaterials, membrane materials, and mesoporous materials, were also applied to soil remediation. In the future, the cost-effective and environmentally friendly methods combined with new technologies might increase the efficiency and sustainability of soil amendment.

### 4.5. Phytoremediation

Soil phytoremediation refers to the utilization of certain heavy metal accumulating plants to reduce the metal content or alleviate the toxic effects in the soils, which is an eco-friendly and sustainable approach to restoring contaminated land. Among various phytoremediation techniques (phytoextraction, rhizofiltration, phytostabilization, and phytovolatilization), phytoextraction is the most efficient method for the removal of heavy metals from soil [97]. The process mechanism of phytoextraction includes (1) the mobilization and migration of heavy metals toward the rhizosphere; (2) the uptake of heavy metals by plant roots; (3) the translocation of heavy metals from root to shoot; (4) the compartmentation of these ions in the plant tissue [98]. Usually, the plants used in this technique have high tolerance to heavy metal stress and can accumulate high metal content in their aerial parts, which are defined as accumulators and hyperaccumulators. Up to 2020, over 750 plant species were reported as hyperaccumulators, and around 70% of them are Ni hyperaccumulators. Some plants that accumulate very high amounts of certain metals have been employed to take up metals in harvestable plant biomass, which is regarded as phytomining; for example, *Phyllanthus balgooyi* is a Ni hyperaccumulator, with the Ni content in its tissue exceeding 10,000 mg kg^−1^, and the Ni in its “bio-ore” (the product of biomass incineration after harvesting) could reach up to 13 wt% [99].

The selection of plants with fast growth and high biomass production rates is critical for phytoremediation. It is reported that some natural heavy metal accumulators or hyperaccumulators are effective in soil remediation, which includes *Thlapsi caeruliensis*, *Pteris vittata* L. (Chinese brake fern), *Sedum alfredii* H., *Brassica juncea* (Indian mustard). For example, *Pteris vittata* L. was found to be highly resistant to As, accumulating 1442–7526 mg kg^−1^ of As in its fronds from contaminated soils, and up to 27,000 mg kg^−1^ of As in its fronds under hydroponic conditions [100]. *Sedum alfredii* H. is a kind of efficient Cd/Zn hyperaccumulator, which can accumulate thousands of mg kg^−1^ Zn and thousands of mg kg^−1^ Cd in the shoots [101]. In addition, some non-hyperaccumulators with high biomass could also be candidate plant species to accumulate high amounts of heavy metals, and intensive planting and harvesting can effectively remove the metal from the soil. 

Phytoremediation accompanied by metal chelating molecules (e.g., EDTA, EGTA, CA), phytohormone (e.g., IAA, ABA, GA), or symbiotic association (e.g., plant growth-promoting rhizobacteria, mycorrhiza) is important in terms of enhancing the remediation efficiency since phytoremediation is a relatively slow process. Chelating molecules such as EDTA could chelate heavy metals via ligand complexes and increase the solubility and translocation of metals from soil to plant roots, consequently improving the phytoextraction efficiency [102]. The application of EDTA significantly increased the bioavailability of Cd in the soil, and about 100% higher Cd was observed in EDTA-treated plants compared to the control [103]. However, the application of EDTA usually causes a negative influence on plant growth; thus, it is necessary to apply suitable growth regulators for the minimization of EDTA toxicity on plant growth and find more environmentally friendly chelating agents. Plant growth-promoting rhizobacteria (PGPR) plays an important role in promoting plant growth and enhancing plant tolerance to heavy metal stress by providing nutrients, producing phytohormones, and altering metal mobility in soil [104]. A study showed that the Cd, Pb, and Zn contents in aboveground *Brassica napus* inoculated with *Enterobacter* sp. JYX7 increased by 32%, 200%, and 28%, respectively [105].

Intercropping or rotating crop plants with hyperaccumulators is a sustainable and promising strategy for removing heavy metals from the soil and reducing heavy metals in agricultural products. It was reported that intercropping maize with *Brassica juncea* L. increased the grain yield of maize by 10% and reduced grain Cd by 41% while increasing the Cd in *Brassica juncea* L. by 50%, compared to monoculture [106]. Crop rotation of high accumulation oilseed rape and low accumulation of rice coupled with superposition measure (soil amendment and foliar inhibitor) enhanced the crop yields, increased the Cd accumulation in non-edible parts of oilseed rape, and reduced Cd in rice; after 3 years of phytoremediation, the removal efficiency of Cd from soils was 7.0% and 7.9%, in two fields, respectively [107]. This comprehensive management can accelerate phytoextraction and ensure food safety simultaneously, which are feasible modes for phytoremediation coupled with argo-production in fields slightly contaminated with heavy metals. 

### 4.6. Standards and Regulations

In addition to the above remediation measures, reducing the sources of contamination is also an important step in controlling heavy metal contamination in agricultural soils, which requires the enforcement of strict environmental protection standards and regulations. In some countries, soil environmental quality standards (Table 2) have been developed based on health and ecological risks in order to control pollution and protect living organisms from the toxicity of heavy metals. And the limitation of heavy metals in agricultural inputs (chemical/organic fertilizers, irrigation, etc.) has been established in some countries; the limits of organic fertilizers are given in Table 3. In addition, stringent regulations/laws should be implemented with regard to the emission sources of heavy metals, such as the mining, smelting, and traffic industries. 

## 5. Conclusions

In this study, current soil control and remediation strategies for heavy metal-contaminated agricultural soils are summarized, including low-metal cultivar selection/breeding, physiological blocking, water management, soil amendment, and phytoremediation. The application of these technologies is relatively mature, but there are some limitations. For example, water management is a practical and inexpensive strategy to lower Cd accumulation in rice, but it is not suitable for upland crops. Soil amendments such as lime are effective in immobilizing Cd in acidic soil but have limited effects in alkaline and neutral soil. Phytoremediation is time-consuming, and the addition of leaching agents can cause secondary pollution. Low-metal cultivar planting is proposed as a practical method to address the low-to-medium heavy metal-contaminated farmland. Due to the diversity of soil types and pollution sources, and the heterogeneity of spatial change of heavy metals in soils, it is difficult to promote a universal and cost-effective method; therefore, it is necessary to develop appropriate measures suitable for the local areas and make the management more precise and effective.

## Figures and Tables

**Table 1:** Amendments for remediation of heavy metal-contaminated soils.

Type	Material	Metal	Mechanism
Lime	CaO, CaCO_3_, Ca(OH)_2_	Cd, Pb, Zn, Cu	Increase soil pH
Phosphate mineral	Phosphate, phosphate rock powder, hydroxyapatite	Cd, Pb, Zn, Cu, Ni, Hg, Cr, As	Electrostatic interaction, ion exchange, surface complexation, precipitation, etc.
Clay mineral	Sepiolite, kaolinite attapulgite, bentonite	Cd, Pb, Zn, Cu	Complexation, lattice diffusion, and isomorphic substitution
Biochar	Wood/crop residues based biochar	Cd, Pb, Zn, Cu	Increase soil pH, cation-π interaction, electrostatic attraction, ion exchange, complexation, and precipitation
Organic fertilizer	Manure, compost	Cd, Pb	Increase soil pH, complexation, adsorption, and precipitation

**Table 2:** Limits of heavy metals in (agricultural) soils set by different countries (mg/kg).

Heavy Metal	The Netherlands ^a^ [108]	UK [109]	Sweden [110]	Canada [111]	USA ^b^ [112]	India [113]	Japan [114]	China ^d^ [115]
Cd	0.6/12	1–8	0.4	1.4	32	3–6	0.01 ^c^	0.3–0.8/1.5–4.0
Hg	—	8		—	—	—	0.0005 ^c^	0.5–3.4/2.0–6.0
As	20/55	20	—	12	18	—	15	20–40/100–200
Pb	50/530	—	80	70	120	250–500	0.01 ^c^	70–240/400–1000
Cr	—	130	120	64	—	—	0.05 ^c^ (Cr^6+^)	150–350/800–1300
Cu	40/190	—	100	63	70	135–270	125	50–200
Zn	140/720	—	350	200	—	300–600	—	60–190
Ni	—	50	—	—	38	—	—	200–300

^a^. Target/intervention value; ^b^. eco-screening value; ^c^. in soil solution (mg/L); ^d^. risk screening/intervention value.

**Table 3:** Limits of heavy metals in organic fertilizers set by different countries (mg/kg).

Heavy Metal	Germany [116]	UK [117]	USA [118]	Japan [119]	China [120]
Cd	1.5	1.5	1.5	1.5	3
Hg	1	1	—	2	2
As	—	—	—	50	15
Pb	150	200	300	100	50
Cr	100	100	100	500	150
Cu	100	200	1500	—	—
Zn	400	400	2800	—	—
Ni	50	50	—	300	—
